# Unlocking tiny titans: 360 view of the quantum dots nanotechnology for dental applications

**DOI:** 10.3389/fdmed.2025.1503057

**Published:** 2025-03-04

**Authors:** Tasneem Alluhaidan, Isadora Martini Garcia, Meghan Alexis, Masoumah Qaw, Fabrício Mezzomo Collares, Mary Ann Williams, Mary Anne S. Melo

**Affiliations:** ^1^ Dental Biomedical Sciences Ph.D. Program, University of Maryland School of Dentistry, Baltimore, MD, United States; ^2^ Department of Preventive Dental Sciences, College of Dentistry, Imam Abdulrahman Bin Faisal University, Dammam, Saudi Arabia; ^3^ Department of Comprehensive Dentistry, University of Maryland School of Dentistry, Baltimore, MD, United States; ^4^ Department of Restorative Dental Sciences, College of Dentistry, Imam Abdulrahman Bin Faisal University, Dammam, Saudi Arabia; ^5^ Department of Dental Materials, School of Dentistry, Federal University of Rio Grande do Sul, Porto Alegre, Brazil; ^6^ Health Sciences and Human Services Library, University of Maryland, Baltimore, MD, United States

**Keywords:** nanoparticles, nanotechnology, dental caries, drug delivery, dental materials

## Abstract

Quantum dots (QDs) nanotechnology has gained significant attention in dentistry due to its unique properties, such as fluorescence, antimicrobial activity, and drug delivery potential. This review aims to identify the dental applications most actively incorporating QD technology and to examine the distinctive properties of QDs within Dentistry. Employing the Arksey and O'Malley five-stage framework, a systematic search was conducted across PubMed, EMBASE, and Scopus databases for English-language publications on QDs in dentistry. Scientific contributions were evaluated by analyzing publication volume, research trends, patents, and key areas of investigation. Of the 1,034 studies initially identified, 71 were fully screened, with 22 meeting the criteria for data extraction. Results showed that antimicrobial properties and bone regeneration are the primary focus areas for QDs in dental materials. Stock solutions and resin composites are the most common materials developed, with the studies primarily targeting ofenhancing antimicrobial capabilities and osteogenesis enhancement. Over the last decade, QDs have demonstrated potential in enhancing drug delivery, antimicrobial efficacy, and optical performance in dental materials. Despite their growing prominence, the clinical translation of QD-based technologies remains limited due to a lack of long-term studies.

## 1 Introduction

**Figure 1:**
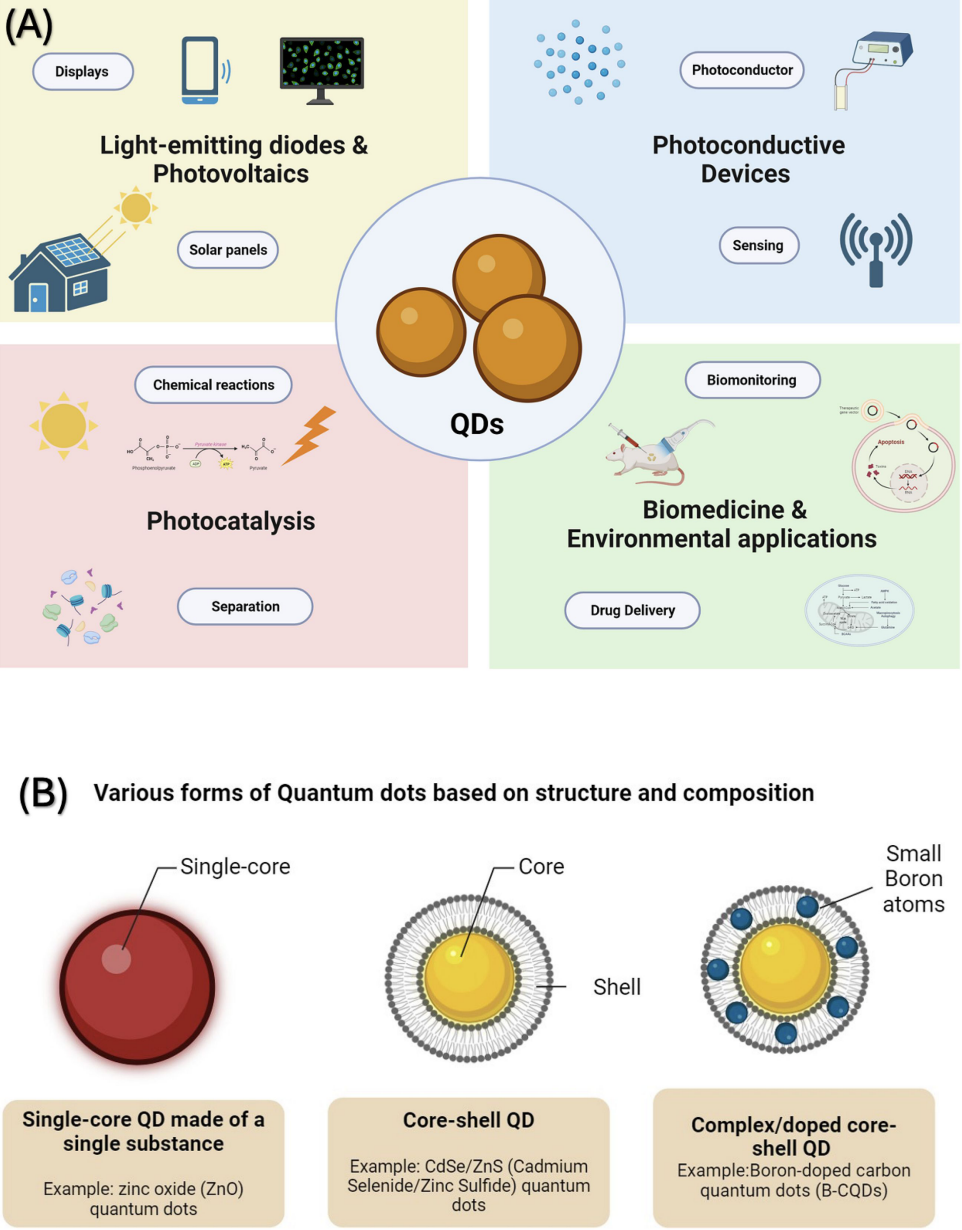
**(A)** Quantum dots are versatile materials used in various technological and medical areas. They are instrumental in creating high-performance lasers and vibrant displays. Additionally, they are key components in solar energy devices currently available on the market. Their applications are expanding into other areas, including photovoltaic systems, sensory technology, bioimaging techniques, targeted drug delivery systems, and quantum information processing. **(B)** Schematic drawing illustrating various forms of Quantum Dots (QDs) based on structure and composition. On the left, show a single-core QD made of a single substance as a simple spherical shape. In the center, illustrate a, with a clear boundary between them. Boron-doped carbon quantum dots (B-CQDs) are displayed on the right, showing a more complex structure with the doping indicated by small boron atoms integrated within the carbon-based lattice.

Nanotechnology is a rapidly advancing field at the cutting edge of science. It holds significant promise for reshaping how we prevent and treat many noncommunicable severe diseases (1). In the rapidly advancing field of biomedicine, the focus on developing nanotechnology-based solutions has gained significant momentum, particularly their potential in precise diagnostics and treatments. It's important to note that Dentistry is also joining this trend, actively seeking advancements to improve oral health (2). This pursuit is powered by the recognition that nanoparticles possess distinctive structural and functional attributes that set them apart from their counterpart's bulk materials (3). Among the most captivating breakthroughs in nanotechnology is the birth of quantum dots (QDs)—a diverse cohort of engineered nanoparticles distinguished for their exceptional optical and chemical characteristics (4). These properties position QDs as pivotal nanoparticles with an expansive spectrum of potential applications, from Medicine to the frontiers of energy exploration (Figure 1A).

Quantum dots are known for their unique physical and chemical characteristics, especially optics (5). Their journey began with their application in biomedical imaging in 1998, which can be traced back to the discovery by Ekimov and Onushenko in 1981, who found quantum dots in a glass matrix (6). In acknowledgment of their groundbreaking work, Ekimov, Brus, and Bawendi were collectively honored with the Nobel Prize in Chemistry on October 4, 2023, for their pioneering work in discovering and synthesizing quantum dots (7). In the last three decades, their uses have diversified, covering areas like solar cells, LEDs, photodetectors, and various computing and biomedical imaging aspects. In biomedical research, quantum dots (QDs) are uniquely suited for cellular biomolecule and organelle tracking due to their diminutive size, which usually spans between 1 and 10 nanometers, holding immense potential for various applications, such as real-time tissue imaging (bioimaging), diagnostics, single molecule probes, and drug delivery, to name a few (8). The unique optical characteristics of these quantum dots can be customized by altering their size and composition. Some notable features include intense luminescence, durability against photobleaching, a substantial surface-to-volume ratio, and antimicrobial activity capable of generating reactive oxygen species (9, 10). These attributes have transformed (11) into valuable applications in biotechnology and Medicine, including Dentistry.

Quantum dots (QDs) come in various forms based on structure and composition. Some consist of a single substance (single-core), while others are made up of two substances (core-shell) or doped like boron-doped carbon quantum dots (B-CQDs) (10) (Figure 1B). This versatility of composition, size, surface properties, and how they interact with bacterial cells provide antibacterial, anti-inflammatory, osteogenic, and other biological effects compelling for investigations with potential applications in several dental disciplines and specialties (12–14). This is particularly significant in Dentistry, where major oral diseases like dental caries and periodontal diseases are biofilm-driven. These conditions are also characterized by inflammatory triggers and are known promoters of bone loss (15). A future dental material incorporating quantum dots (QDs) with antibacterial and antibiofilm properties could be highly beneficial in preventing the recurrence of caries around restorations made with QD-enhanced materials (11).

Understanding the properties of quantum dots in this context is crucial for developing targeted dental treatments. As quantum dot technology gains attraction within Dentistry, the current stage and specific application dimensions require clarification. Numerous publications have explored various topics related to Quantum Dots (QDs), encompassing their latest developments, analysis of their structure–activity relationships, ways they interact with cells, and their uses in the medical field (9, 16, 17). However, there's a noticeable gap in the literature regarding a comprehensive examination of QDs' applications in Dentistry. This includes an in-depth look at their typical chemical compositions, the challenges they present, and their future potential within this specific area of use. Therefore, this review aims to comprehensively assess quantum dots' current status and applications in dentistry, capitalizing on their potential role in oral healthcare.

Our objective is to provide an overview of the extensive range of applications, global presence, and research caliber, thereby creating a comprehensive map of the quantum dot landscape within the dental field.

## 2 Materials and methods

### 2.1 Materials study and design

A scoping review is an effective method for exploring the use of quantum dots in dentistry and identifying under-researched areas. The current study aimed to gather and evaluate the latest evidence on applying quantum dots in dental practices. In line with the guidelines provided by Levac, Colquhoun, O'Brien and the framework of Arskey and O'Malley, we employed a five-stage process for this review. These stages included defining the research questions, identifying relevant studies, selecting the appropriate ones, charting the data, and compiling the findings for reporting.

### 2.2 Stage I: identification of research question

This section outlines the research methodology and the specific research questions: “*What is the current scope and extent of research on the applications of quantum dots in dentistry, including their usage, benefits, challenges, and future prospects*?”

### 2.3 Stage II: identification of eligible studies

Under the supervision of a research librarian (M.A.W), a comprehensive search was conducted in databases including OVID Medline, EMBASE, and SCOPUS. This search focused exclusively on English-language articles, imposing no restrictions on the year of publication. The latest actualization was in June 2024, where we identified all relevant studies on the subject. The search strategy involved querying terms in the titles, abstracts, and keywords sections. These terms were customized for each database using the Boolean operators “AND” and “OR.” As described ((“quantum dots” OR “quantum dot” OR “QDs” OR “QD”) AND (“dentistry” OR “dental” OR “tooth” OR “teeth” OR “oral health” OR “oral” OR “dental materials” OR “dental material” OR “caries” OR “streptococcus mutans” OR “S. mutans”) were the search terms used to find the articles. Additionally, a manual search supplemented this approach. Following the removal of duplicate entries, three review authors (TA, MA, and IG) independently screened each study, focusing on titles, abstracts, and keywords for potential inclusion. Those studies fulfilling the inclusion criteria were then fully analyzed. The authors resolved any disagreements through a joint decision-making process.

### 2.4 Stage III: selection of studies

The inclusion criteria for the studies during stages II and III are (1) The study must specifically investigate the use of quantum dots in dentistry. This includes but is not limited to, applications in dental diagnostics, treatment procedures, imaging techniques, and material science; (2) Studies designed as experimental studies, observational studies, and clinical trials; and (3) Only peer-reviewed studies will be included to ensure the quality and reliability of the data. The following exclusion criteria were applied: (1) Articles mentioning quantum dots but not providing specific data or detailed analysis of their application in dentistry.; (2) Articles published in languages other than English, as these could present challenges in accurate interpretation and analysis; (3) Reviews, Case reports, and grey literature, including non-peer-reviewed articles, conference abstracts, editorials, and commentaries, which may lack the necessary scientific rigor and validation.

### 2.5 Stage IV: data charting

Data extraction was developed in Microsoft Excel and underwent a review by all the authors involved in the study to establish and document the necessary variables for effective data extraction. Subsequently, two authors, TA and IG, were tasked to extract data from the selected studies.

### 2.6 Stage V: extraction of data and result reporting

The data was extracted using a Microsoft® Excel form version 2016. The primary information extracted was (1) Authors and Year of Publication: To identify the study and track its temporal relevance; (2) Study Location: To understand the geographical distribution and context of the research; (3) Quantum Dot Characteristics: Details about the quantum dots used, including their composition, size, synthesis method, and functional properties; (4) Type of Dental Application: Specific dental application where quantum dots are applied (e.g., imaging, diagnostics, treatment); (5) Type of Dental Specialty: Specific dental area of interest where quantum dots are applied (e.g., general dentistry, operative dentistry, prosthodontics, etc); (6) Safety and Biocompatibility: Information on the safety profile and biocompatibility of quantum dots used in dental applications, including any reported adverse effects; (7) Findings and Outcomes: Key results of the study, focusing on the effectiveness, benefits, and limitations of quantum dots in dental applications.

## 3 Results

### 3.1 Study selection

**Figure 2:**
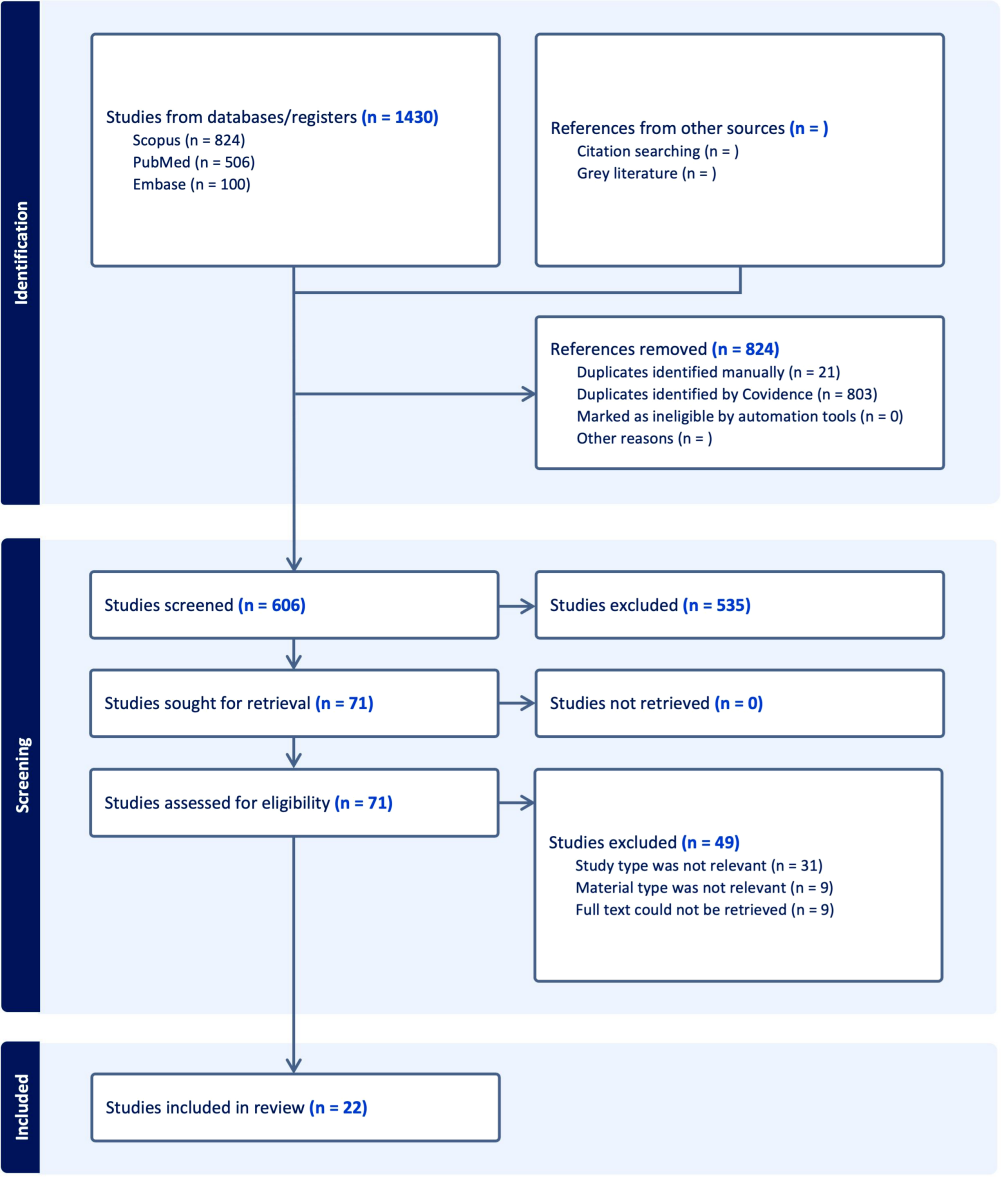
PRISMA flowchart of the study outlines the study selection process for the systematic review, showing the steps from initial identification of studies to final inclusion.

Searches ended on June, 2024, with 1,430 studies imported for screening. Figure 2 shows a PRISMA flowchart with the search and selection process details. After deleting duplicates, 606 studies were selected for title and abstract screening. Full-text screening was permitted for 71 records. After a full-text review, only 22 studies were relevant, leaving 22 for content analysis.

### 3.2 Global trends and focus areas

**Figure 3:**
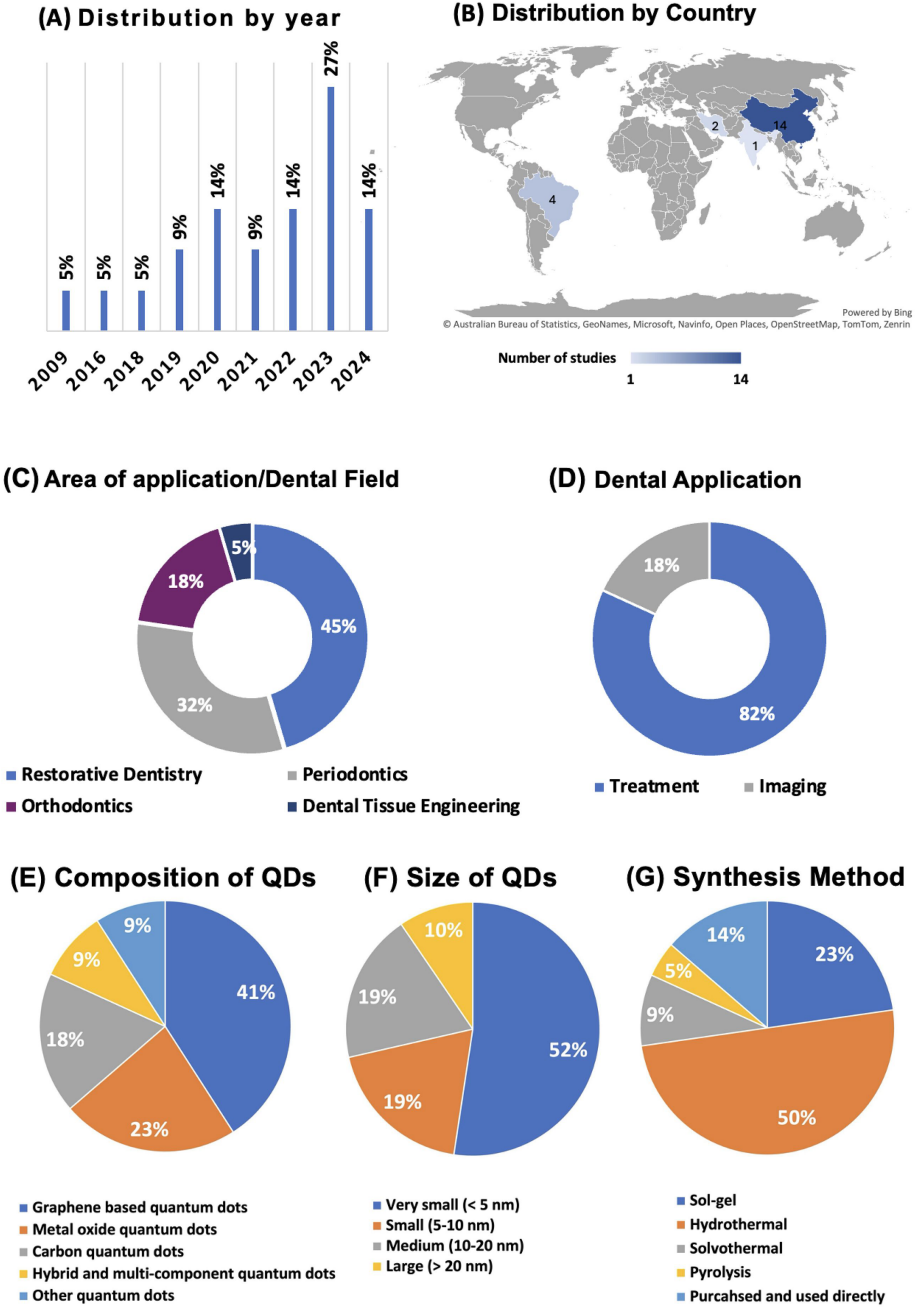
Overview of global trends and studies distributions. **(A)** Displays the yearly distribution of studies, highlighting a significant rise in publications in 2023; **(B)** shows the geographical distribution of studies, with China and Brazil contributing the most research; **(C)** divides studies based on dental applications, with the majority focusing on treatment, and **(D)** categorizes the research by dental field, with a strong emphasis on restorative dentistry. **(E)** The composition of quantum dots used in dental studies, with graphene-based dots leading; **(F)** displays the size distribution, with the majority of QDs being very small (<5 nm); **(G)** Summarizes the synthesis methods, showing hydrothermal as the most common technique.

Figure 3 displays the global trends and areas of investigations of QDs in Dentistry. In Figure 3A, the distribution of dental studies by year shows a steady increase in the number of studies conducted from 2009 to 2024. Notably, there was a constant rate of studies at 5% from 2009 until 2018. A notable increase began in 2019, stabilizing at 9% until 2022, before surging to 14% in both 2023 and 2024. This indicates a growing interest and focus in dental research investigating QDs. Figure 3B shows the geographical distribution and highlights that most research is concentrated in three countries. China leads with 14 studies, followed by Brazil, India, and Iran. This geographical distribution underscores the significant research output from the United States in dentistry.

The majority of the studies (82%) focused on treatment applications in dentistry, while a smaller portion (18%) addressed imaging technologies (Figure 3C). This suggests a predominant research interest in developing and enhancing dental treatment methodologies. Regarding the areas of application, QD studies spanned various dental fields with a predominant focus on Restorative Dentistry, which accounted for 45% of the research. This was followed by Periodontics and Orthodontics, which represented 32% and 18% of the studies, respectively. Dental Tissue Engineering was the least studied area, making up only 5% of the total. This distribution strongly emphasizes restorative practices, reflecting ongoing efforts to improve and innovate in restorative dental materials (Figure 3D).

### 3.3 QDs composition, average size, and synthesis

Figure 3E illustrates the percentage distribution of QDs based on their composition. The largest proportion of QDs belongs to the *Graphene-Based Quantum Dots* category, accounting for 41% of the total compositions. *Metal Oxide Quantum Dots* make up 23%, reflecting their widespread use in various applications. The *Carbon Quantum Dots* category represents 18% of the total, while *Other Quantum Dots* and *Hybrid and Multi-Component Quantum Dots* each represent 9% of the distribution. These findings indicate a strong focus on graphene-based QDs, likely due to their favorable electrical and optical properties. Figure 3F highlights the size distribution of the QDs. The majority, 52%, fall under the *Very Small* category (less than 5 nm in size), indicating a trend toward developing extremely small QDs for applications that require nanoscale materials, such as bioimaging and drug delivery. The *Small* (5–10 nm) and *Medium* (10–20 nm) size categories each represent 19% of the distribution, while the *Large* QDs (greater than 20 nm) make up 10% of the total. This size distribution suggests a preference for very small QDs in dental research, where surface area and quantum effects are critical for performance.

*Hydrothermal* synthesis is the most common method, accounting for 50% of the total, reflecting its effectiveness in producing high-quality QDs. *Sol-gel* synthesis follows, representing 23% of the total, a method known for its simplicity and versatility in forming QD structures (Figure 3G). The purchased and used category directl*y* accounts for 14%, indicating that some of the QDs used in research are commercially available. *Solvothermal* and *Pyrolysis* methods contribute 9% and 5%, respectively, representing less frequently used but still significant approaches to QD synthesis. This panel underscores the prominence of hydrothermal and sol-gel methods in QD fabrication.

### 3.4 Investigated properties and dental materials developed incorporating QDs

**Figure 4:**
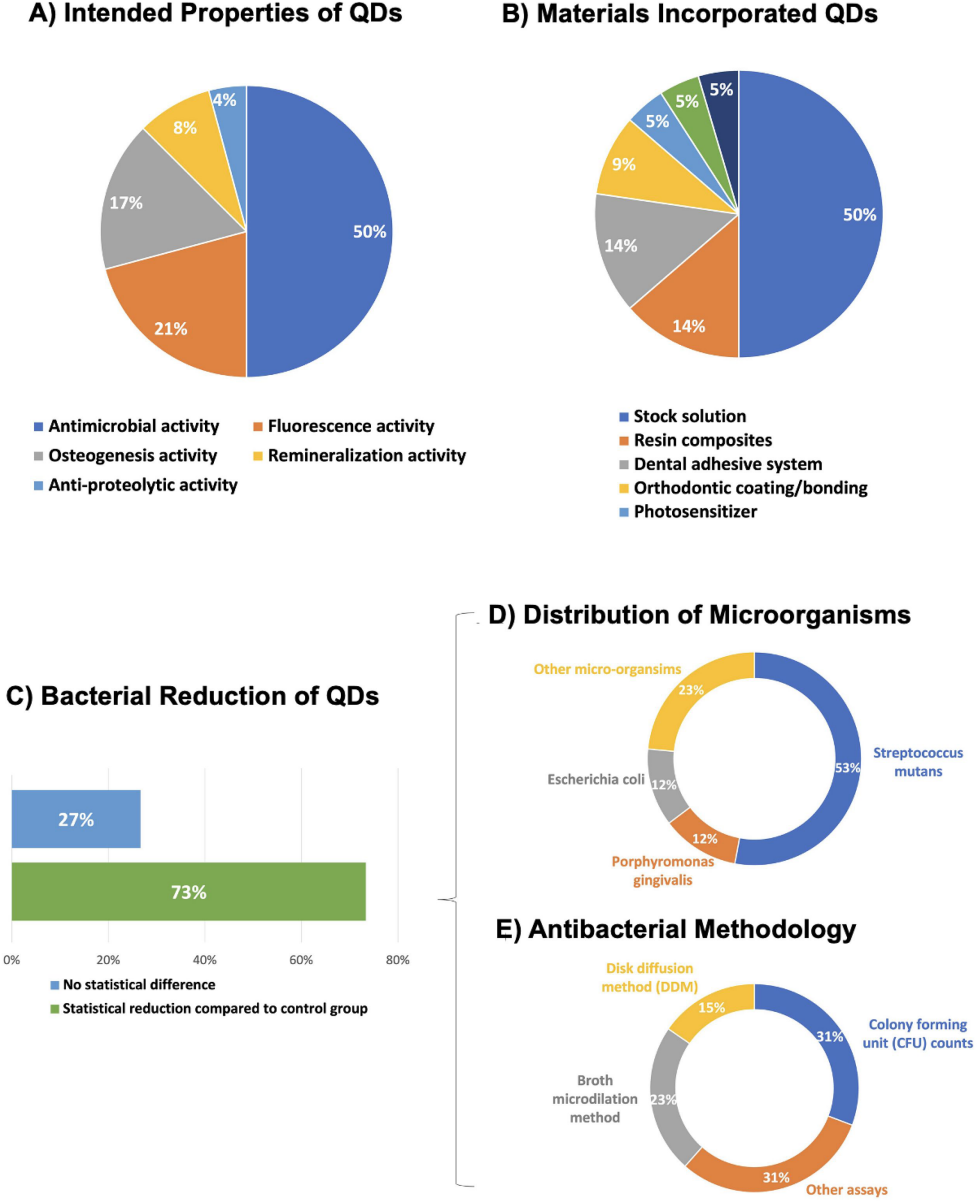
**(A)** Focuses on the investigated properties of QDs, with antimicrobial activity being the primary focus; **(B)** shows the types of materials incorporating QDs, with stock solutions being the most frequent material. **(C)** Percentage of QD's studies where the main outcome of the antimicrobial assays was statistically superior and similar to a control group. **(D)** The distribution of microorganisms tested. Streptococcus mutans, a major cariogenic pathogen, accounts for 53% of the total tested bacteria. Porphyromonas gingivalis and Escherichia coli each represents 12%, with the remaining 23% composed of other microorganisms. **(E)** Methods used to evaluate antibacterial activity. Colony Forming Unit (CFU) counts and other assays account for 31% each, while broth microdilution and disk diffusion methods (DDM) make up 23% and 15%, respectively.

The primary goals of the studies involving QDs are summarized in Figure 4A. Most of the research aimed at conveying antibacterial activity by incorporating QDs, representing 50% of the total, showcasing the emphasis on preventing bacterial infections and promoting oral health. Fluorescence activity is the second most explored property, representing 21%, highlighting the potential for QDs to be used in diagnostic applications. Osteogenesis activity is the third common property, representing 17%, indicating the possibility of QDs to promote bone regeneration. Lastly, remineralization accounts for 5%, suggesting using QDs to reinforce tooth enamel and dentin structures.

Types of materials that incorporate QDs are illustrated in Figure 4B. The largest category is Stock Solution, comprising 50%, showing that QDs are often studied dispersed in solution form. Resin Composite accounts for 14%, demonstrating its intense investigations in conjunction with QDs for develop antibacterial resin composites. Dental Adhesive System follows at 14%. Other categories include Orthodontic Coating/Bonding (9%), Photosensitizer (5%), Dental Cement (5%), and Glass Ionomer Cement (5%), which indicates diverse applications of QDs across different dental materials.

In further consideration of the prevailing target involving QDs to convey antibacterial activity, 73% of main outcome for QDs' studies has shown statistical bacterial reduction compared to a control group (Figure 4C). The outcomes of the remaining studies have demonstrated no significant difference (27%).

**Figure 5:**
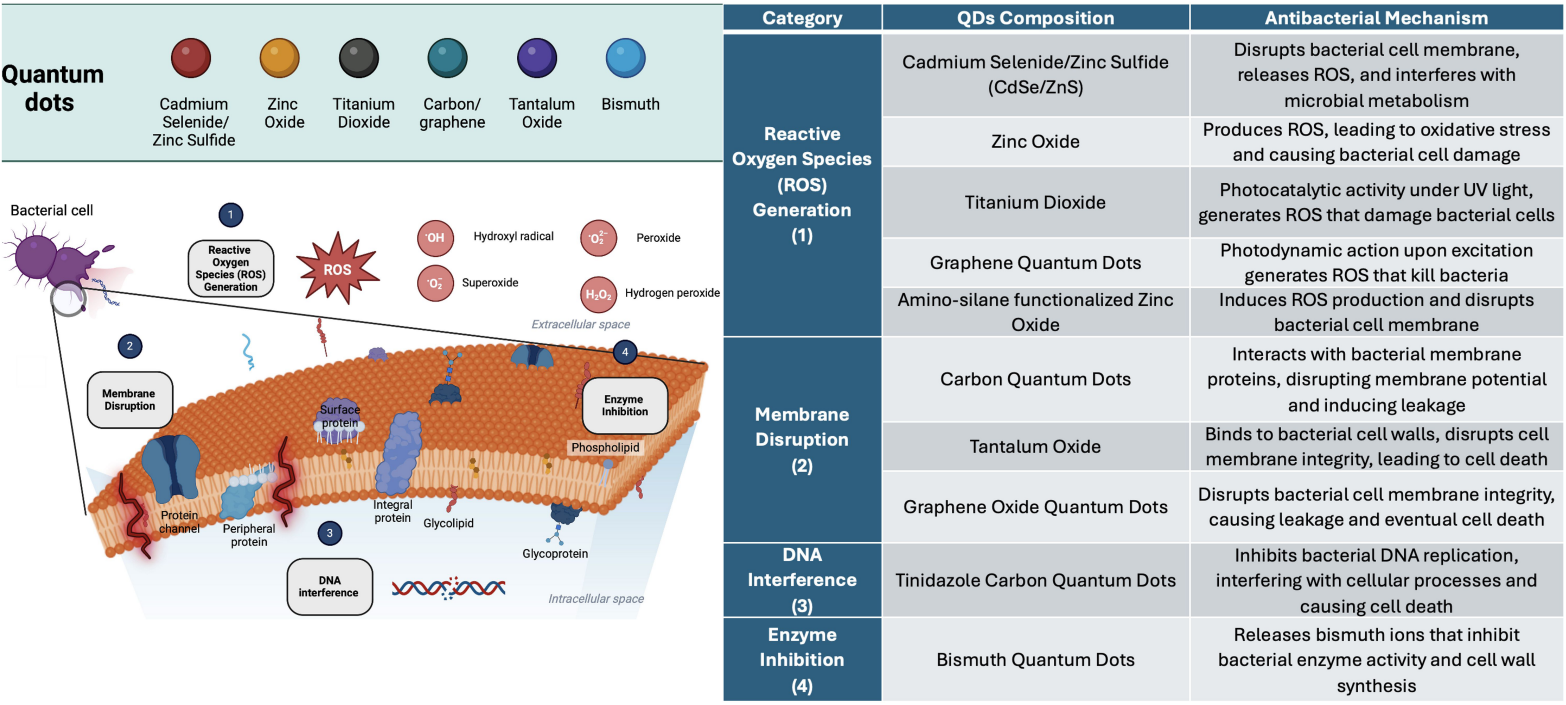
The antibacterial mechanisms of various quantum dots (QDs), categorized into four primary modes of action: reactive oxygen Species (ROS) generation, membrane disruption, DNA interference, and enzyme inhibition. Reactive Oxygen Species (ROS) Generation (1): QDs such as Cadmium Selenide/Zinc Sulfide (CdSe/ZnS), Zinc Oxide, Titanium Dioxide, and Graphene Quantum Dots induce oxidative stress by producing ROS (e.g., hydroxyl radicals, superoxide, hydrogen peroxide), which damage bacterial proteins, lipids, and DNA. Membrane Disruption (2): Carbon Quantum Dots, Tantalum Oxide, and Graphene Oxide Quantum Dots disrupt the integrity of the bacterial cell membrane by interacting with proteins and lipids, leading to cell leakage and death. DNA Interference (3): Tinidazole Carbon Quantum Dots disrupt bacterial DNA replication, interfering with essential cellular processes and leading to cell death. Enzyme Inhibition (4): Bismuth Quantum Dots release ions that inhibit bacterial enzyme activity, particularly enzymes involved in cell wall synthesis, weakening the bacteria and causing cell death.

In relation to the distribution of microorganisms tested. *Streptococcus mutans*, a major cariogenic pathogen, constituted 53% of the bacteria examined. *Porphyromonas gingivalis* and *Escherichia coli* both represented 12%, while other microorganisms accounted for the remaining 23% (Figure 4D). The methodologies used to assess the antibacterial activity are detailed in Figure 4E. Colony Forming Unit (CFU) and other assays (31% each) were the most frequently used methods. Broth microdilution and disk diffusion methods (DDM) contributed 23% and 15% of the assays, respectively, highlighting the diversity of techniques used to evaluate antibacterial efficacy. Figure 5 illustrates the antibacterial mechanisms of quantum dots (QDs).

Table 1 shows the results from the cytotoxicity and biocompatibility assessments of various quantum dots (QDs), demonstrating consistent findings across multiple studies. Most QDs showed no significant cytotoxic effects compared to control groups under the tested conditions. For example, studies using human gingival fibroblasts (HGFs), including those by Garcia et al. (18), Pourhajibagher et al. (19), Cao et al. (25), and Hosseinpour-Nader et al. (27), reported no statistically significant cytotoxic effects. These studies utilized Sulforhodamine B colorimetric and MTT assays, supporting that QDs can be safely applied in dental-related contexts with minimal adverse cellular effects. Similarly, in Liang et al. (20), evaluations using the MTT assay and hemolysis assay on human immortalized liver cells (L0–2) and rat red blood cells (RBCs) revealed no toxic effects up to concentrations of 100 μg/ml, indicating a low systemic toxicity risk for these quantum dots. Furthermore, the stem cells from human exfoliated deciduous teeth (SHEDs) tested in Yang et al. (21) showed no cytotoxic effects up to 50 μg/ml, further highlighting the potential of QDs for dental and regenerative applications. In periodontal ligament fibroblasts (PDLFs) and periodontal ligament stem cells (PDLSCs), the findings of Hu et al. (22) and Liang et al. (26) indicated no cytotoxicity at concentrations up to 800 ppm and 50 μg/ml, respectively, reinforcing the safety of QDs in periodontal treatment settings.

**Table 1:** Table: overview of the cytotoxicity and biocompatibility assessments of various quantum dots (QDs) and their outcomes. This table provides a summary of different studies evaluating the cytotoxicity and biocompatibility of quantum dots (QDs) using various assays and cell types. Each entry details the author, the year of the study, the specific assays employed (e.g., MTT, CCK-8, Sulforhodamine B colorimetric assay), the types of cells tested (e.g., human gingival fibroblasts, stem cells from human exfoliated deciduous teeth), and the key outcomes. Across multiple studies, the results show that most quantum dots did not exhibit significant cytotoxic effects compared to control groups, supporting their biocompatibility under the tested conditions.

Author	Cytotoxicity/biocompatibility assays	Cells used	Main outcome of cytotoxicity/biocompatibility assays
Garcia et al. (18)	Cytotoxicity: Sulforhodamine B colorimetric assay	Human gingival fibroblasts (HGFs)	No statistical effects compared to control group.
Pourhajibagher et al. (19)	Cytotoxicity: MTT assay	Human gingival fibroblasts (HGFs)	No statistical effects compared to control group.
Liang et al. (20)	Cytotoxicity: MTT assay Biocompatibility/hemocompatibility: Hemolysis assay	Human immortalized liver cell line (L0–2) Rat red blood cells (RBCs)	No statistical effects compared to control group up to 100 μg/ml.
Yang et al. (21)	Cytotoxicity: CK-8 assay	Stem cells from human exfoliated deciduous teeth (SHEDs)	No statistical effects compared to control group up to 50 μg/ml.
Hu et al. (22)	Cytotoxicity: CCK-8 assay Cell live/dead staining (Calcein-AM/PI)	Periodontal ligament fibroblasts (PDLFs) Periodontal ligament stem cells (PDLSCs)	No statistical effects compared to control group up to 800 ppm.
Yan et al. (23)	Cytotoxicity: CCK-8 assay Cell live/dead staining (Calcein-AM/PI) Histocompatibility: Subcutaneous tissue contact test	Human gingival fibroblasts (HGFs) Subcutaneous cells	No statistical effects compared to control group.
Lu et al., (24)	Cytotoxicity: Cell live/dead staining (Calcein-AM/PI) MTT assay	Osteoblasts	Statistical reduction compared to control group from 0.5–1.0 wt.% GQD.
Cao et al. (25)	Cytotoxicity: CCK-8 assay Cell live/dead staining (Calcein-AM/PI)	Human gingival fibroblasts (HGFs)	No statistical effects compared to control group.
Liang et al. (26)	Cytotoxicity: CCK-8 assay	Periodontal ligament stem cells (PDLSCs)	No statistical effects compared to control group.
Hosseinpour-Nader et al. (27)	Cytotoxicity: Neutral red assay	Human gingival fibroblasts (HGFs)	No statistical effects compared to control group.
Lin et al. (28)	Cytotoxicity: CCK-8 assay	Dental pulp cells (DPSCs)	No statistical effects compared to control group until concentration reached 50 μg/ml.
Xue et al. (29)	Cytotoxicity: CCK-8 assay Cell live/dead staining (Calcein-AM/PI)	Hepatocyte growth factor (HGF)	No statistical effects compared to control group.
Zhang et al. (41)	Biocompatibility: Immunofluorescence staining assay	Bone marrow mesenchymal stem cells (BMSCs)	No statistical effects compared to control group.
An et al. (30)	Cytotoxicity: CCK-8 assay	Human periodontal ligament stem cells (hPDLSCs)	No statistical effects compared to control group up to 5 μg/ml of Y-GOQds and up to 50 μg/ml of B-GOQDs.
Josephraj et al. (31)	Cytotoxicity: MTT assay	Human dental pulp stem cells (hDPSCs)	No statistical effects compared to control group up to 0.125 mg ml^−1^ concentrations.
Jiang et al. (11)	Cytotoxicity: CCK-8 assay Cell live/dead staining (Calcein-AM/PI) Biocompatibility/hemocompatib-ility: Hemolysis assay	Macrophages Rat red blood cells (RBCs)	No statistical effects compared to control group.

## 4 Discussion

This review highlights the escalating interest and diversity in applying Quantum Dots (QDs), a field at the cutting edge of nanotechnology in dentistry. Designing new composite materials involves integrating various components to enhance properties such as antibacterial, durability, and biocompatibility (32). The studies analyzed demonstrate a broad spectrum of chemical compositions in QDs, including zinc oxide, graphene oxide, tantalum oxide, and titanium oxide. This variety underscores the adaptability of QDs, allowing customization for specific dental applications. The predominant use of graphene oxide is particularly notable, suggesting its potential as a versatile and practical component in dental materials.

Graphene Quantum Dots (GQD) have emerged as a predominant choice in dental applications due to their unique properties that align well with the requirements of dental materials (33). Firstly, GQDs possess exceptional mechanical strength and stability, which are essential in the oral environment where materials are subject to constant mechanical stress. This durability ensures dental materials incorporated with GQDs can withstand biting forces and abrasive actions, maintaining their integrity over time (34). Additionally, GQDs exhibit remarkable biocompatibility, which is critical in avoiding adverse reactions in sensitive oral tissues (22, 34). GQDs also have antimicrobial properties, providing an added advantage in preventing and treating dental infections. Their ability to inhibit the growth of common oral pathogens, such as *Streptococcus mutans*, is particularly valuable in addressing dental caries and other microbial-related oral diseases (24). Furthermore, GQDs are relatively easier and cost-effective to synthesize compared to other quantum dots, making them more accessible for research and application in dental materials (35). The combination of these properties positions GQDs as a versatile and effective material in advancing dental technology, justifying their predominant use in current research and applications (35). Graphene-based QDs have shown lower cytotoxicity levels, indicating a safer profile for dental applications (36).

The review's outcome also reveals a significant focus on the antimicrobial properties of QDs against Streptococcus mutans, a key factor in developing dental caries. This focus aligns with the essential need for effective antimicrobial agents in dentistry (12). QD-containing-adhesives, representing a major research avenue, were primarily evaluated for their antimicrobial effects and physical-mechanical properties, reflecting their potential in restorative dentistry (37). Geographical analysis of the studies indicates a concentrated effort in countries like Brazil (18, 37–39) and China (11, 20–30, 40–42). This distribution may mirror specific regional research interests or resource allocations in nanotechnology and dental research.

When examining QDs as isolated compounds, the studies broadly explore the impact of chemical composition on cytotoxicity and physical-chemical properties, with graphene oxide again emerging as a dominant component. Bismuth quantum dots were explored in the remaining 25%, indicating the ongoing exploration of different QD compositions for diverse dental applications (22). The focus on interdisciplinary dentistry, endodontics, and periodontics illustrates the wide-ranging applicability of QD-based materials across dental specialties.

The interdisciplinary dentistry field showed a substantial inclination towards incorporating QDs into adhesive resins, constituting approximately 83% of the studies. This trend underscores the cross-disciplinary potential of QD-based dental materials, with implications for restorative and preventive dentistry. Incorporating QDs into orthodontic appliances and bioactive glass opens new research avenues (23, 40). While the studies in orthodontic applications delve into antimicrobial efficacy, particularly against S. mutans, the solitary study in bioactive glass investigates its enhanced physical and chemical properties (43). These findings hint at the potential for QDs to innovate beyond conventional dental materials.

Despite its comprehensive nature, this review has limitations. Firstly, the scope of included studies, confined to articles published between January 2013 and June 2024, may omit relevant earlier research. Secondly, the focus on English-language publications potentially overlooks significant contributions in other languages. This may influence the findings by excluding relevant research published in different languages, which could provide valuable insights or alternative perspectives. Furthermore, excluding grey literature, such as conference papers and technical reports, might lead to an incomplete understanding of the current landscape of QD research in dentistry. Finally, the review's dependence on published studies may introduce publication bias, as studies with positive results are more likely to be published than those with negative or inconclusive findings.

In summary, the exploration of QDs in dentistry is marked by a dynamic research landscape with diverse applications and geographic contributions. However, the limitations of this review should be considered when interpreting the findings and planning future research directions in this innovative are of interest. Overall, the diversity of applications, chemical compositions, and geographic distribution in the reviewed studies highlights the expanding frontier of QD-based dental research. The emphasis on antimicrobial effects, physical properties, and chemical compositions reflects the multifaceted potential of QDs in advancing various aspects of dentistry, from restorative materials to orthodontic appliances and beyond. Future research should focus on standardizing the synthesis of QDs, exploring their multifunctional capabilities, and assessing their long-term safety and translation for clinical settings. Additionally, investigating their integration with other nanomaterials could pave the way for innovative target disease approaches.

## 5 Conclusion

The results of the reviewed studies indicate a growing interest in utilizing Quantum Dots (QDs) within dentistry, with various applications and chemical compositions being explored. The QD chemical composition displayed a wide range, with multiple compounds such as zinc oxide, graphene oxide, tantalum oxide, and titanium oxide mainly being investigated. The diversity in chemical compositions suggests a dynamic approach to tailoring QD properties for specific dental applications using graphene oxide as the most prevalent chemical composition.

## References

[1] Bhatia SN Chen X Dobrovolskaia MA Lammers T. Cancer nanomedicine. Nat Rev Cancer. (2022) 22(10):550–6. 10.1038/s41568-022-00496-9 35941223 PMC9358926

[2] Jandt KD Watts DC. Nanotechnology in dentistry: present and future perspectives on dental nanomaterials. Dent Mater. (2020) 36(11):1365–78. 10.1016/j.dental.2020.08.006 32981749 PMC7516471

[3] Melo MAS Guedes SFF Xu HHK Rodrigues LKA. Nanotechnology-based restorative materials for dental caries management. Trends Biotechnol. (2013) 31(8):459–67. 10.1016/j.tibtech.2013.05.010 23810638 PMC3845439

[4] Tajik S Dourandish Z Zhang K Beitollahi H Le QV Jang HW Carbon and graphene quantum dots: a review on syntheses, characterization, biological and sensing applications for neurotransmitter determination. RSC Adv. (2020) 10(26):15406–29. 10.1039/D0RA00799D 35495425 PMC9052380

[5] Cotta MA. Quantum dots and their applications: what lies ahead? ACS Appl Nano Mater. (2020) 3(6):4920–4. 10.1021/acsanm.0c01386

[6] Ekimov AI Efros A Onushchenko AA. Quantum size effect in semiconductor microcrystals. Solid State Commun. (1985) 56(11):921–4. 10.1016/S0038-1098(85)80025-9

[7] Manna L. The bright and enlightening science of quantum dots. Nano Lett. (2023) 23(21):9673–6. 10.1021/acs.nanolett.3c03904 37870455 PMC10636900

[8] Filali S Pirot F Miossec P. Biological applications and toxicity minimization of semiconductor quantum dots. Trends Biotechnol. (2020) 38(2):163–77. 10.1016/j.tibtech.2019.07.013 31473014

[9] Boopathy LK Gopal T Roy A Kalari Kandy RR Arumugam MK. Recent trends in macromolecule-conjugated hybrid quantum dots for cancer theranostic applications. RSC Adv. (2023) 13(27):18760–74. 10.1039/D3RA02673F 37346950 PMC10281231

[10] Wagner AM Knipe JM Orive G Peppas NA. Quantum dots in biomedical applications. Acta Biomater. (2019) 94:44–63. 10.1016/j.actbio.2019.05.022 31082570 PMC6642839

[11] Jiang Y Hua Z Geng Q Li N. Carbon quantum dots carrying antibiotics for treating dental implant bacterial infections following photothermal therapy. Nano. (2024) 19(01):2450004. 10.1142/S1793292024500048

[12] Rajendiran K Zhao Z Pei DS Fu A. Antimicrobial activity and mechanism of functionalized quantum dots. Polymers. (2019) 11(10):1670. 10.3390/polym11101670 31614993 PMC6835343

[13] Rosenthal SJ Chang JC Kovtun O McBride JR Tomlinson ID. Biocompatible quantum dots for biological applications. Chem Biol. (2011) 18(1):10–24. 10.1016/j.chembiol.2010.11.013 21276935 PMC3752999

[14] Gao W Liang Y Wu D Deng S Qiu R. Graphene quantum dots enhance the osteogenic differentiation of PDLSCs in the inflammatory microenvironment. BMC Oral Health. (2023) 23(1):331. 10.1186/s12903-023-03026-7 37244994 PMC10225102

[15] Bertolini M Costa RC Barão VAR Villar CC Retamal-Valdes B Feres M Oral microorganisms and biofilms: new insights to defeat the main etiologic factor of oral diseases. Microorganisms. (2022) 10(12):2413. 10.3390/microorganisms10122413 36557666 PMC9781395

[16] Kunachowicz D Ściskalska M Jakubek M Kizek R Kepinska M. Structural changes in selected human proteins induced by exposure to quantum dots, their biological relevance and possible biomedical applications. NanoImpact. (2022) 26:100405. 10.1016/j.impact.2022.100405 35560289

[17] Belal F Mabrouk M Hammad S Ahmed H Barseem A. Recent applications of quantum dots in pharmaceutical analysis. J Fluoresc. (2023) 34(1):119–38. 10.1007/s10895-023-03276-2 37222883

[18] Garcia IM Souza VS Hellriegel C Scholten JD Collares FM. Ionic liquid–stabilized titania quantum dots applied in adhesive resin. J Dent Res. (2019) 98(6):682–8. 10.1177/0022034519835203 30905311

[19] Pourhajibagher M Parker S Chiniforush N Bahador A. Photoexcitation triggering via semiconductor graphene quantum dots by photochemical doping with curcumin versus perio-pathogens mixed biofilms. Photodiagnosis Photodyn Ther. (2019) 28:125–31. 10.1016/j.pdpdt.2019.08.025 31479805

[20] Liang G Shi H Qi Y Li J Jing A Liu Q Specific anti-biofilm activity of carbon quantum dots by destroying P. gingivalis biofilm related genes. Int J Nanomedicine. (2020) 15:5473–89. 10.2147/IJN.S253416 32801701 PMC7406331

[21] Yang X Zhao Q Chen J Liu J Lin J Lu J Graphene oxide quantum dots promote osteogenic differentiation of stem cells from human exfoliated deciduous teeth via the wnt/β-catenin signaling pathway. Stem Cells Int. (2021) 2021:8876745. 10.1155/2021/8876745 33628273 PMC7886518

[22] Hu Y Xu Z Hu Y Hu L Zi Y Wang M Bismuth quantum dot (Bi QD)/polydimethylsiloxane (PDMS) nanocomposites with self-cleaning and antibacterial activity for dental applications. Nanomaterials. (2022) 12(21):3911. 10.3390/nano12213911 36364687 PMC9656007

[23] Yan J Hua F Cao L Yang H He H. Multifunctional modification of orthodontic adhesives with ZnO quantum dots. Dent Mater. (2022) 38(11):1728–41. 10.1016/j.dental.2022.09.003 36137833

[24] Lu S Zhang H Chai M Yao X Zhang X Yang Y. Mechanical and antibacterial properties of resin co-filled with mesoporous silica and graphene quantum dots. Carbon Lett. (2023) 33(2):373–85. 10.1007/s42823-022-00426-7

[25] Cao L Yan J Luo T Yan H Hua F He H. Antibacterial and fluorescent clear aligner attachment resin modified with chlorhexidine loaded mesoporous silica nanoparticles and zinc oxide quantum dots. J Mech Behav Biomed Mater. (2023) 141:105817. 10.1016/j.jmbbm.2023.105817 37015147

[26] Liang Y Gao W Deng S Wu D Jiang Y Zhang Y Graphene quantum dots promote migration and differentiation of periodontal ligament stem cells. Front Chem. (2023) 11:1213507. 10.3389/fchem.2023.1213507 38025053 PMC10679356

[27] Hosseinpour-Nader A Karimi N Ghafari HA. Ex-vivo effects of propolis quantum dots-nisin-nanoquercetin-mediated photodynamic therapy on Streptococcus mutans biofilms and white spot lesions. Photodiagnosis Photodyn Ther. (2023) 41:103255. 10.1016/j.pdpdt.2022.103255 36567010

[28] Lin L Zheng Y Wang C Li P Xu D Zhao W. Concentration-dependent cellular uptake of graphene oxide quantum dots promotes the odontoblastic differentiation of dental pulp cells via the AMPK/mTOR pathway. ACS Omega. (2023) 8(6):5393–405. 10.1021/acsomega.2c06508 36816699 PMC9933470

[29] Xue J Dong H Ji L Wang Y Zhang J. Peptide-functionalized ZnSe:Mn quantum dots as fluorescent probes for accurate localization of hidden dental lesion sites. ACS Appl Nano Mater. (2023) 6(15):14431–8. 10.1021/acsanm.3c02446

[30] An N Yan X Qiu Q Zhang Z Zhang X Zheng B Human periodontal ligament stem cell sheets activated by graphene oxide quantum dots repair periodontal bone defects by promoting mitochondrial dynamics dependent osteogenic differentiation. J Nanobiotechnol. (2024) 22(1):133. 10.1186/s12951-024-02422-7 PMC10976692 38539195

[31] Josephraj F Kumar N A Nandini V V Karthik SS. Performance evaluation of carbon quantum dots impregnated glass ionomer cement to avoid peri-implant disease. Biomed Mater. (2024) 19(3):035040. 10.1088/1748-605X/ad407b 38636498

[32] Alluhaidan T Qaw M Garcia IM Montoya C Orrego S Melo MA. Seeking endurance: designing smart dental composites for tooth restoration. Designs. (2024) 8(5):92. 10.3390/designs8050092

[33] Williams AG Moore E Thomas A Johnson JA. Graphene-based materials in dental applications: antibacterial, biocompatible, and bone regenerative properties. Int J Biomater. (2023) 2023:1–18. 10.1155/2023/8803283 PMC9929215 36819211

[34] Cui Y Liu L Shi M Wang Y Meng X Chen Y A review of advances in graphene quantum dots: from preparation and modification methods to application. Journal Carbon Res. (2024) 10(1):7. 10.3390/c10010007

[35] Dananjaya V Marimuthu S Chunhui YR Grace AN Abeykoon C. Synthesis, properties, applications, 3D printing and machine learning of graphene quantum dots in polymer nanocomposites. Prog Mater Sci. (2024) 144:101282. 10.1016/j.pmatsci.2024.101282

[36] Li X Liang X Wang Y Wang D Teng M Xu H Graphene-based nanomaterials for dental applications: principles, current advances, and future outlook. Front Bioeng Biotechnol. (2022) 10:804201. 10.3389/fbioe.2022.804201 35360406 PMC8961302

[37] Garcia IM Souza VS Scholten JD Collares FM. Quantum dots of tantalum oxide with an imidazolium ionic liquid as antibacterial agent for adhesive resin. J Adhes Dent. (2020) 22(2):207–14. 10.3290/j.jad.a44285 32322841

[38] Garcia IM Leitune VCB Kist TL Takimi A Samuel SMW Collares FM. Quantum dots as nonagglomerated nanofillers for adhesive resins. J Dent Res. (2016) 95(12):1401–7. 10.1177/0022034516656838 27422857

[39] Alves LP Pilla V Murgo DOA Munin E. Core-shell quantum dots tailor the fluorescence of dental resin composites. J Dent. (2010) 38(2):149–52. 10.1016/j.jdent.2009.09.014 19804811

[40] Zhang J An X Li X Liao X Nie Y Fan Z. Enhanced antibacterial properties of the bracket under natural light via decoration with ZnO/carbon quantum dots composite coating. Chem Phys Lett. (2018) 706:702–7. 10.1016/j.cplett.2018.06.029

[41] Zhang X-y Lu S-x He D-m Chai M-z Wu Z-z Yao X-h Antibacterial property of graphene quantum dots-modified TiO_2_ nanorods on titanium dental implant. Trans Nonferrous Met Soc China. (2023) 33(8):2395–405. 10.1016/S1003-6326(23)66267-3

[42] Chen W Jin H Zhang H Wu L Chen G Shao H Synergistic effects of graphene quantum dots and carbodiimide in promoting resin-dentin bond durability. Dent Mater. (2021) 37(10):1498–510. 10.1016/j.dental.2021.07.004 34465445

[43] Son SA Kim DH Yoo KH Yoon SY Kim YI. Mesoporous bioactive glass combined with graphene oxide quantum dot as a new material for a new treatment option for dentin hypersensitivity. Nanomaterials. (2020) 10(4):621. 10.3390/nano10040621 32230884 PMC7221916

